# Antiviral Treatment against Monkeypox: A Scoping Review

**DOI:** 10.3390/tropicalmed7110369

**Published:** 2022-11-10

**Authors:** Brando Ortiz-Saavedra, Darwin A. León-Figueroa, Elizbet S. Montes-Madariaga, Alex Ricardo-Martínez, Niza Alva, Cielo Cabanillas-Ramirez, Joshuan J. Barboza, Abdelmonem Siddiq, Luis A. Coaguila Cusicanqui, D. Katterine Bonilla-Aldana, Alfonso J. Rodriguez-Morales

**Affiliations:** 1 Facultad de Medicina, Universidad Nacional de San Agustín de Arequipa, Arequipa 04000, Peru; 2 Facultad de Medicina Humana, Universidad de San Martín de Porres, Chiclayo 14012, Peru; 3 Unidad de Revisiones Sistemáticas y Meta-Análisis, Tau-Relaped Group, Trujillo 13001, Peru; 4 Escuela de Medicina, Universidad Peruana de Ciencias Aplicadas, Lima 15023, Peru; 5 Vicerrectorado de Investigación, Universidad Norbert Wiener, Lima 15023, Peru; 6 Faculty of Pharmacy, Mansoura University, Mansoura 35516, Egypt; 7 Facultad de Medicina Humana, Universidad Peruana Antenor Orrego, Trujillo 13001, Peru; 8 Research Unit, Universidad Continental, Huancayo 12001, Peru; 9 Grupo de Investigación Biomedicina, Faculty of Medicine, Fundacion Universitaria Autonoma de las Americas, Pereira 660001, Risaralda, Colombia; 10 Latin American Network of MOnkeypox VIrus Research (LAMOVI), Pereira 660001, Risaralda, Colombia; 11 Master of Clinical Epidemiology and Biostatistics, Universidad Cientifica del Sur, Lima 15023, Peru; 12 Gilbert and Rose-Marie Chagoury School of Medicine, Lebanese American University, Beirut P.O. Box 36, Lebanon

**Keywords:** monkeypox, monkeypox virus, antiviral treatment, orthopoxvirus, scoping review

## Abstract

During the COVID-19 pandemic, the increase in reports of human monkeypox virus infection cases spreading in many countries outside Africa is a major cause for concern. Therefore, this study aimed to explore the evidence of antiviral pharmacotherapy available for the treatment of adult patients with monkeypox. A scoping review of the literature was conducted using PubMed, Scopus, Web of Science, Embase, and CENTRAL databases until 12 September 2022. The key search terms used were “monkeypox” and “treatment”. A total of 1927 articles were retrieved using the search strategy. After removing duplicates (*n* = 1007) and examining by title, abstract, and full text, 11 studies reporting case reports of monkeypox with antiviral treatment were included, detailing the number of monkeypox cases, clinical manifestations, number of participants with antiviral treatment, history of sexually transmitted diseases, method of diagnosis, location of skin lesions, drugs used in antiviral treatment, route of administration, and outcome. A total of 1281 confirmed cases of monkeypox have been reported, of which 65 monkeypox cases had antiviral treatment distributed most frequently in the United States (*n* = 30), the United Kingdom (*n* = 6), and Spain (*n* = 6). Of the total cases, 1269 (99.1%) were male with an age range of 18 to 76 years, and 1226 (95.7%) had a sexual behavior of being men who have sex with men. All confirmed cases of monkeypox were diagnosed by reverse transcriptase polymerase chain reaction (RT-PCR). The most frequent clinical manifestations were skin lesions, fever, lymphadenopathy, headache, fatigue, and myalgia. The most frequent locations of the lesions were perianal, genital, facial, and upper and lower extremities. The most commonly used drugs for antiviral treatment of monkeypox were: tecovirimat, cidofovir, and brincidofovir. All patients had a complete recovery. According to current evidence, the efficacy and safety of antiviral drugs against monkeypox is of low quality and scarce.

## 1. Introduction

During the COVID-19 pandemic, increased reporting of human monkeypox virus infection cases spreading in many countries outside of Africa is a major cause for concern [1]. As of 21 October 2022, 75,348 confirmed cases of monkeypox (MPX) have been reported in 109 countries worldwide [2].

MPX is a zoonotic viral disease caused by the monkeypox virus (MPXV) [3]. MPXV is a double-stranded DNA virus of the genus Orthopoxvirus of the family Poxviridae known for more than half a century but geographically restricted to a limited number of endemic countries in Central and West Africa [4].

The transmission of MPX to humans occurs mainly through contact with body fluids, skin lesions, or respiratory droplets from animals infected directly or indirectly through contaminated fomites [5]. The symptoms are similar to those of the disease known as smallpox, with MPX usually involving fever, a skin rash that is more common on the face and extremities than on the trunk, and lymphadenopathy, which is a characteristic feature of MPX [6], with an incubation period varying from 5 to 21 days [7].

MPXV has two distinct genetic clades: the Central African clade (Congo Basin) and the West African clade [8]. The West African clade is known to have a more favorable prognosis, with a case fatality rate of less than 1%. On the other hand, the Central Basin clade (Central African clade) is more lethal, with a case fatality rate of up to 10% in unvaccinated children [9].

At present, there is no authoritative treatment or adequate evidence-based guideline for the treatment of MPX. Thus, clinical management aims to provide symptomatic treatment, manage complications, and prevent long-term sequelae [4]. Although MPX is usually self-limiting and does not require medical treatment, there are some antiviral treatment alternatives, such as tecovirimat, brincidofovir (BCV), and cidofovir, which have been approved in animal models, but their efficacy has not been measured in humans [10,11]. However, the United States has recommended a licensed vaccine, JYNNEOS (Smallpox and MPX Vaccine, Live, Nonreplicating) for the vaccination of persons at risk of occupational exposure to Orthopoxviruses [12].

Although current vaccines offer cross-protection against MPX, they are not specific against the disease-causing MPXV, and their efficacy has not yet been proven in light of recent outbreaks in several countries [13]. Moreover, as a consequence of the eradication and cessation of smallpox vaccination for four decades, MPXV found an opportunity to re-emerge, but with different characteristics [14].

The objective of the present scoping review is to explore the evidence on antiviral pharmacotherapy available for the treatment of adult patients with MPX.

## 2. Materials and Methods

### 2.1. Protocol and Registration

The recommendations of the Preferred Reporting Items for Systematic and Meta-Analysis Extension for Scoping Reviews (PRISMA-ScR) [15] and the methodological criteria of the Joana Briggs Institute were followed in the present scoping review [16]. The protocol was previously registered in the Figshare platform (https://doi.org/10.6084/m9.figshare.20577312.v1) (accessed on 31 August 2022).

### 2.2. Eligibility Criteria

We included primary research articles on patients over the age of 18 who had a serological diagnosis, Polymerase Chain Reaction (PCR), electron microscopy, or immunohistochemical findings positive for MPX and who received some type of pharmacological treatment with an antiviral mechanism of action. The types of studies included in the present review were case reports, case series, observational studies (cohort, cross-sectional, case-control), and clinical trials (randomized and non-randomized). No language limit was established for the articles, and publications were included until 12 September 2022. Scoping reviews, systematic reviews, narrative reviews, letters to the editor without original results, and conference proceedings and abstracts were excluded.

### 2.3. Information Sources and Search Strategy

A systematic search was carried out in Pubmed, Embase, Scopus, Web of Science, and the Cochrane Controlled Trials Register (CENTRAL). The search terms used were: “Monkeypox” and “treatment” (Table 1). The searches were completed on 12 September 2022, and four different investigators independently evaluated the search results.

### 2.4. Study Selection

Three investigators (B.O.-S., D.A.L.-F., and E.S.M.-M.) created a database based on the electronic searches, managed with the appropriate management software (EndNote (Clarivate Analytics, Philadelphia, PA, the United States)), and duplicates were removed. Then, through Rayyan QCRI [17], three researchers (A.M., J.J.B., and N.A.) carried out the screening process, analyzing the titles and abstracts provided by the search independently, choosing those that appeared to meet the inclusion criteria and, if necessary, evaluating the full text. In case of disagreement, the investigators will discuss it until a consensus is reached; in case of a dispute, a fourth investigator will be invited to the discussion to help resolve it.

The authors (A.S., L.A.C.C., D.K.B.-A., and A.J.R.-M.) reviewed the full-text reports and analyzed the inclusion criteria to reach a decision.

### 2.5. Outcomes

The primary outcome was to report on the antiviral drug therapy available for the treatment of adult patients with MPX.

### 2.6. Data Collection Process and Data Items

Three investigators independently extracted data from the selected studies into a Microsoft Excel spreadsheet. The following data were extracted from the selected studies: First author, country, year of publication, study design, number of patients, age, sex, method of MPX diagnosis, clinical signs, comorbidities, period of illness, antiviral treatment, dose, mode of administration, discharge, and death. A fourth investigator checked the list of articles and data extractions to ensure that there were no duplicate articles or duplicate information and resolved discrepancies about study inclusion. The results are summarized in narrative form and tables.

## 3. Results

### 3.1. Study Selection

**Figure 1:**
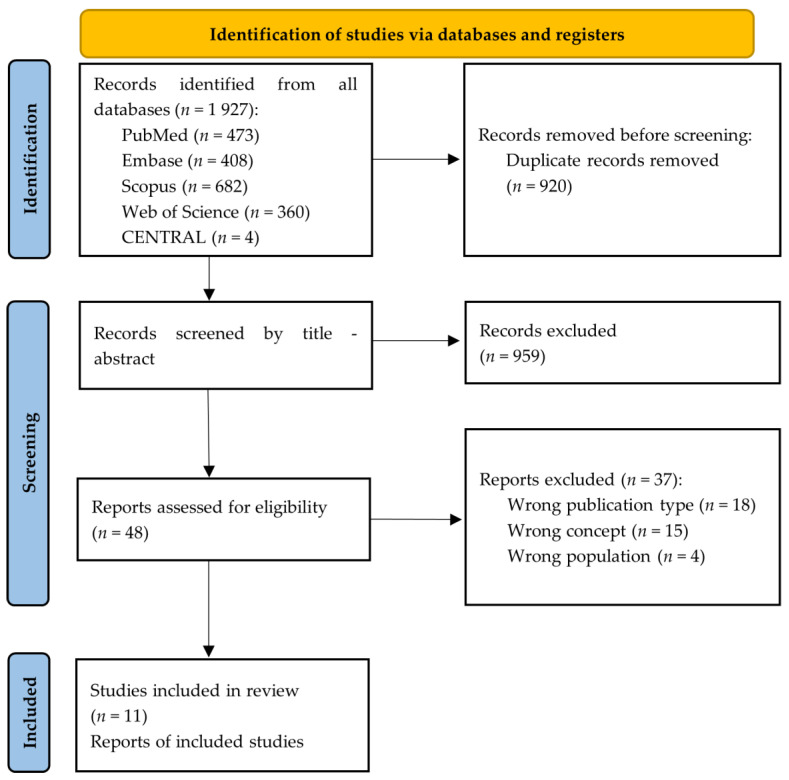
PRISMA flowchart summarizing the study selection process.

A total of 1927 articles were retrieved using the search strategy. The selection strategy is shown in the prism flow chart (Preferred Reporting Items for Systematic and Meta-Analysis Extension for Scoping Reviews). After the removal of duplicates (*n* = 920), 1007 articles were screened by the reviewers. After filtering the titles and reading the abstracts, 48 articles were selected for full-text reading, and 11 were considered eligible for inclusion in this Scoping Review (Figure 1) [1,18,19,20,21,22,23,24,25,26,27].

### 3.2. Study Characteristics

The main characteristics of the articles included in this scoping review are summarized in Table 2 [1,18,19,20,21,22,23,24,25,26,27]. Our scoping review included 11 studies that were published between 1 January and 12 September 2022. The included studies (*n* = 11) reported case reports of MPX applying antiviral treatment, detailed the number of MPX cases, clinical manifestations, number of participants with antiviral treatment, history of sexually transmitted diseases, method of diagnosis, location of skin lesions, drugs used in antiviral treatment, route of administration, and outcome (Table 2 and Table 3) [1,18,19,20,21,22,23,24,25,26,27]. A total of 1281 confirmed MPX cases were reported [1,18,19,20,21,22,23,24,25,26,27], of which 65 MPX cases had antiviral treatment [1,18,19,20,21,22,23,24,25,26,27] distributed in the United States (*n* = 30) [19,20,22,24], the United Kingdom (*n* = 6) [18,25,27], Spain (*n* = 6) [26], Italy (*n* = 1) [21], France (*n* = 1) [23], and Others (*n* = 21) [1] (Table 1). The United States was the country with the highest number of MPX cases receiving antiviral treatment, followed by the United Kingdom and Spain.

### 3.3. Demographical Characteristics and Diagnostic Method for Monkeypox

Of the total number of cases (*n* = 1281) reported with MPX [1,18,19,20,21,22,23,24,25,26,27], 1269 (99.1%) cases were male [1,18,19,20,21,22,23,24,25,26,27]. The age range of reported MPX cases was 18 to 76 years [1,18,19,20,21,22,23,24,25,26,27]. Of the reported cases with MPX, 1226 (95.7%) had the sexual behavior of being men who have sex with men [1,18,19,20,21,22,23,24,25,26,27]. The sexually transmitted diseases reported in MPX patients were gonorrhea (*n* = 89) [1,25,27], syphilis (*n* = 66) [1,25,27], chlamydia (*n* = 43) [1,26,27], and herpes simplex (*n* = 38) [1,25,27], and 458 patients tested positive for Human Immunodeficiency Virus (HIV) [19,20,21,23,25,26,27]. All confirmed cases of MPX were diagnosed by reverse transcriptase polymerase chain reaction (RT-PCR) [1,18,19,20,21,22,23,24,25,26,27] (Table 2).

### 3.4. Clinical Manifestations, Localization of Skin Lesions, and Treatment

The most frequent clinical manifestations in patients confirmed with MPX were skin lesions (*n* = 1253) [1,18,19,20,21,22,23,24,25,26,27], fever (*n* = 821) [1,18,19,20,21,22,23,24,25,26,27], lymphadenopathy (*n* = 800) [1,18,19,20,21,23,24,25,26,27], headache (*n* = 397) [1,18,19,20,21,23,24,26,27], myalgia (*n* = 246) [1,19,20,25,27] and fatigue (*n* = 103) [1,19,22,24,25,27] (Table 3). The most frequent locations of lesions were the genital area (*n* = 797) [1,18,19,20,21,23,24,25,26,27], trunk (*n* = 508) [1,18,21,23,24,26,27], upper and lower extremities (*n* = 557) [1,18,19,20,21,23,24,25,26,27], face (*n* = 379) [1,18,19,21,23,24,25,27], and perianal area (*n* = 302) [1,19,21,23,24,25,26,27] (Table 3). The most commonly used drugs for antiviral treatment of MPX were tecovirimat (*n* = 41) [1,18,19,20,22,23,24,25,27], cidofovir (*n* = 20) [1,21,23,26], and BCV (*n* = 3) [18] (Table 3). The route of administration for tecovirimat was oral, for cidofovir it was parenteral and topical, and for BCV oral [1,18,19,20,21,22,23,24,25,26,27]. In addition, some patients reported adverse effects such as headache, fatigue, nausea, and transaminitis [1,18,19,20,21,22,23,24,25,26,27]. The majority of patients did not refer to a specific treatment but limited themselves to following the treatments for the sexually transmitted diseases they were suffering from. No deaths were reported; all patients made a full recovery [1,18,19,20,21,22,23,24,25,26,27].

## 4. Discussion

There is currently no approved treatment specifically for MPXV infections [28]. However, antivirals developed for use in smallpox patients may be beneficial against MPX, therefore the objective of the present scoping review is to explore the evidence on antiviral pharmacotherapy available for the treatment of adult patients with MPX. It is important to know the correct management of these patients to help mitigate the disease process in order to avoid possible sequelae and even death.

In total, we included 11 studies involving 1281 patients, with the United Kingdom being the country with the most reported patients. All confirmed cases of MPX were diagnosed by RT-PCR. Most of the patients were male and stated that they had the sexual behavior of being men who have sex with men. This would indicate the importance of the sexual transmission mechanism in the spread of MPX. In addition, 35.7% of patients tested positive for HIV, which could predispose them to a more aggressive development of MPX disease.

According to the World Health Organization, reports that current epidemiological statistics indicate that young males are disproportionately affected, with 98.2% (20,138/20,500) of patients with gender information being male and a median age of 36 years (interquartile range: 30–43 years). A total of 95.8% (9484/9899) of patients with reported sexual orientation were identified as males who have intercourse with men [29].

It was reported that only 5% of patients received antiviral treatment against MPX, most often with tecovirimat or cidofovir. Although there is a lack of information on these substances’ efficacy in humans, animal research and case studies imply that they may be effective [30].

Tecovirimat (ST-246) is an antiviral drug that was approved by the Food and Drug Administration (FDA) for the treatment of smallpox disease. Tecovirimat has activity against orthopoxviruses but no notable activity against other dsDNA viruses [31]. One of the main targets of tecovirimat is the palmitoylated phospholipase F13 or p37. F13 is located in the viral envelope and membrane and is involved in the formation of the extracellular enveloped virus (EVV). EVV is hypothesized to be a major contributor to viral entry, cell-to-cell transmission, and transmission through the bloodstream to distant tissues [31,32,33]. In the present review, 41 participants received tecovirimat at a dose of 600 mg twice daily for 2 weeks. All participants recovered, while the most frequent adverse effects were fatigue, headache, and nausea. In addition, some patients developed transaminitis. Clinical trials were previously conducted on healthy volunteers, where they evaluated the safety and pharmacokinetics of tecovirimat [34,35]. In general, adverse effects were mild and did not leave sequelae, the most frequent being headache and nausea [30]. The efficacy of tecovirimat was previously demonstrated in animal models [30,36], but no data are yet available in humans. A multicenter Phase 2 clinical trial evaluated the safety, tolerability, and pharmacokinetics of tecovirimat when administered as a single daily oral dose (400 mg or 600 mg) for 14 days in adult volunteers 18 to 74 years of age and found it to be safe and well tolerated, with no deaths or serious adverse events [35]. Two Phase III clinical trials are currently underway to evaluate the efficacy of tecovirimat for the treatment of MPX (NCT05534984, NCT05534165), both with tentative completion dates of 2023.

Cidofovir is a drug used to treat poxviruses; however, the FDA has only approved its usage in Acquired Immune Deficiency Syndrome (AIDS) patients for cytomegalovirus retinitis. Cidofovir diphosphate (CDV-pp), a prodrug that enters cells and is phosphorylated to its active form by cellular enzymes, has the effect of integrating into the developing DNA strand and slowing DNA synthesis. It can also decrease DNA polymerase’s 3′-5′ exonuclease activity [31]. It has been demonstrated to be effective in treating molluscum contagiosum lesions in individuals with AIDS-related CMV retinitis [37]. It is also effective against bovine smallpox and Tecovirimat-associated eczema [38,39]. There is inadequate clinical evidence to support the efficacy of cidofovir against MPX in humans. However, its in vitro efficacy has been demonstrated on MPX infections in animals [10,40,41]. A clinical trial evaluated the synthesis and in vitro and in vivo activity of cidofovir against a variety of orthopoxviruses, it was found to be a broad-spectrum antiviral with potent activity against orthopoxviruses [42].

BCV is another treatment that was approved by the FDA to treat smallpox in 2021. This drug has shown efficacy in treating other orthopoxviruses. BCV, a lipid conjugate of cidofovir, is an acyclic lipid nucleoside phosphonate that is available orally. Unlike cidofovir, BCV provides fewer toxic effects, such as nephrotoxicity, which has been demonstrated after intravenous (IV) dosing in animals and humans and has benefits such as oral use [31,43]. BCV shows activity against several DNA viruses, mainly the poxviruses as the MPXV [44]. It was evidenced in a study in which 3 MPX patients were treated with a weekly BCV dose of 200 mg, that it demonstrated a reduction of the viral load; however, none of the patients completed the treatment because they reported elevations of hepatic enzymes, without observing other hematological alterations [18]. A clinical trial evaluated the safety of brincidofovir against orthopoxvirus in healthy adult subjects and found it to be safe and well tolerated, with no serious or life-threatening adverse reactions [45]. In addition, a Phase II clinical trial evaluating the use of BCV IV in patients with adenovirus infection is currently underway (NCT04706923). Importantly, clinical trials are still needed to evaluate the efficacy and safety of BCV in MPX.

The situation of this re-emerging zoonotic disease is very worrying and deserves further study on the antiviral treatments that can be used against this disease that is currently affecting several continents and with possible new routes of transmission, even during the COVID-19 pandemic that has not yet ended. Key interventions to prevent MPX outbreaks include a high index of suspicion, early identification, isolation, barrier nursing, and strict infection prevention practices by healthcare workers [46].

### Limitations and Strengths

Among the limitations of this scoping review is the small number of eligible participants who received antiviral therapy against MPX. In addition, most of the studies corresponded to case reports and case series, where there was no control group with which to compare the effectiveness of the antiviral drugs used. Therefore, with the current evidence, it is not possible to provide a conclusion regarding the efficacy of antiviral drugs. Future randomized clinical studies with appropriate methods are required to assess the effectiveness of tecovirimat, BCV, cidofovir, and other drugs, particularly in populations thought to be at risk, such as HIV patients. In addition, another limitation is that we did not identify articles from the gray literature. In terms of strengths, the present study has a rigorous methodology since it was conducted by the JBI and PRISMA-ScR guidelines. Likewise, all the processes carried out for the selection of studies were performed independently by two or more authors.

## 5. Conclusions

MPX has spread rapidly throughout the world, and there is less information on the effectiveness of antiviral drugs in treating this disease. However, because smallpox and MPXV are genetically related, antiviral drugs developed to protect against smallpox could also be used to treat MPXV infections. Patients with compromised immune systems and those who are more likely to develop the severe disease may be advised to take antivirals, such as tecovirimat. According to current studies, symptoms usually resolve with or without treatment; however, future randomized clinical trials are needed to determine the efficacy and safety of tecovirimat, cidofovir and BCV against MPX.

## Figures and Tables

**Table 1:** Bibliographic search strategy.

Base	Search Strategy
PUBMED	#1 “Monkeypox” [MH] OR “Monkeypox virus” [MH] OR “Monkeypox” [TIAB] OR “Monkey Pox” [TIAB] OR “Monkeypoxvirus*” [TIAB] #2 “Therapeutics” [MH] OR “Therapeutic Uses” [MH] OR “Therap*” [TIAB] OR “Treatment*” [TIAB] OR “Pharmaco*” [TIAB] OR “Antiviral*” [TIAB] OR “Management*” [TIAB] OR “Drug*” [TIAB] OR “Agent*” [TIAB] #3 = #1 AND #2
SCOPUS	#1 TITLE-ABS-KEY (“Monkeypox” OR “Monkeypox virus” OR “Monkey Pox” OR “Monkeypoxvirus*”) #2 TITLE-ABS-KEY (“Therap*” OR “Treatment*” OR “Pharmaco*” OR “Antiviral*” OR “Management*” OR “Drug*” OR “Agent*”) #3 = #1 AND #2
WEB OF SCIENCE	#1 ALL = (“Monkeypox” OR “Monkeypox virus” OR “Monkey Pox” OR “Monkeypoxvirus*”) #2 ALL = (“Therap*” OR “Treatment*” OR “Pharmaco*” OR “Antiviral*” OR “Management*” OR “Drug*” OR “Agent*”) #3 = #1 AND #2
EMBASE	#1 ‘monkeypox’/exp OR ‘monkeypox’ #2 ‘therapy’ #3 = #1 AND #2
CENTRAL	#1 “Monkeypox” OR “Monkeypox virus” OR “Monkey Pox” OR “Monkeypoxvirus*” #2 “Therap*” OR “Treatment*” OR “Pharmaco*” OR “Antiviral*” OR “Management*” OR “Drug*” #3 = #1 AND #2

**Table 2:** Characteristics of included studies.

Authors	Year	Design	Country	Number of Patients (*n*)	Age (Years)	Sex (M/F)	Sexual Behavior	Previous STIs	HIV Status	Diagnostic Method for Monkeypox	Patients Who Received Antiviral Treatmen (*n*)
Adler H, et al. [18]	2022	Case series	United Kingdom	1	Range (30–40)	M	NR	None	Negative	RT-PCR	*n* = 4
				2	Range (30–40)	M	NR	None	Negative	RT-PCR	
				3	Range (30–40)	F	NR	None	Negative	RT-PCR	
				4	Range (41–50)	M	NR	None	Negative	RT-PCR	
				5	Range (30–40)	M	NR	None	Negative	RT-PCR	
				6	Range (<2)	F	NR	None	Negative	RT-PCR	
				7	Range (30–40)	F	NR	None	Negative	RT-PCR	
Desai AN, et al. [19]	2022	Case series	United States	25	Median = 40.7 (26–76)	M (*n* = 25)	MSM (*n* = 25)	NR	Positive (*n* = 9)	RT-PCR	*n* = 25
Matias WR, et al. [20]	2022	Case series	United States	1	20	M	MSM	Gonococcal urethritis	Negative	RT-PCR	*n* = 3
				2	20	M	MSM	HIV	Positive	RT-PCR	
				3	40	M	MSM	None	Negative	RT-PCR	
Thornhill JP, et al. [1]	2022	Case series	16 countries	528	Median: 38 (18–68)	M (*n* = 527) F (*n* = 0) Trans (*n* = 1)	Heterose-xual (*n* = 9) Homose-xual (*n* = 509) Bisexual (*n* = 10)	STI (*n* = 377) Gonorrhea (*n* = 32/377), Chlamydia (*n* = 20/377), Syphilis (*n* = 33/377), Herpes simplex (*n* = 3/377), Lympho-granuloma venereum (*n* = 2/377), Chlamydia and gonor-rhea (*n* = 5/377), Other or not stated (*n* = 14/377)	Positive (*n* = 218)	RT-PCR	*n* = 21
Moschese D, et al. [21]	2022	Case series	Italy	1	26	M	NR	NR	Negative	RT-PCR	*n* = 1
				2	35	M	NR	NR	Negative	RT-PCR	
				3	34	M	NR	NR	Positive	RT-PCR	
				4	37	M	NR	NR	Positive	RT-PCR	
Rao AK, et al. [22]	2022	Case report	United States	1	NR	M	Hetero-sexual	None	None	RT-PCR	*n* = 1
Mailhe M, et al. [23]	2022	Cohort study	France	264	Median: 35 (30–41)	M (*n* = 262) F (*n* = 1) Trans (*n* = 1)	MSM (*n* = 245)	STI (*n* = 209)	Positive (*n* = 73)	RT-PCR	*n* = 1
Minhaj, F.S. et al. [24]	2022	Case reports	United States	17	Median: 40 (28–61)	M (*n* = 17)	GBMSM (*n* = 17)	NR	NR	RT-PCR	*n* = 1
Girometti, N. et al. [25]	2022	Cohort study	United Kingdom	54	Median: 41 (34–45)	M (*n* = 54)	MSM (*n* = 54)	HIV (*n* = 13), Syphilis (*n* = 14), Herpes simplex (*n* = 24) and Gonorrhea (*n* = 13)	Positive (*n* = 13)	RT-PCR	*n* = 1
Tarín-Vicente, E.J. et al. [26]	2022	Cohort study	Spain	181	Median: 37 (31–42)	M (*n* = 175) F (*n* = 6)	MSM (*n* = 166) MSW (*n* = 15)	HIV (*n* = 72), Syphilis (*n* = 13), Chlamydia (*n* = 10)	Positive (*n* = 72)	RT-PCR	*n* = 6
Patel, A. et al. [27]	2022	Case report	United Kingdom	197	Median: 38 (32–42)	M (*n* = 197)	MSM (*n* = 197)	HIV (*n* = 70), Gonorrhea (*n* = 43/161), Chlamydia (*n* = 13/161), Syphilis (*n* = 6/163), Herpes simplex (*n* = 11/157)	Positive (*n* = 70)	RT-PCR	*n* = 1

MSM:  men who have sex with men; MSW: men who have sex with women; GBMSM: gay or bisexual or other men who have sex with men; STI: sexually transmitted infection; HIV: human immunodeficiency virus; RT-PCR: Polymerase chain reaction with reverse transcriptase; M/F: Male/Female; NR: No report.

**Table 3:** Characteristics of eligible studies. Clinical manifestations, localization, the evolution of lesions, and treatment of monkeypox cases.

Authors	Number of Patients (*n*)	Clinical Manifestations	Localization of Skin Lesions	Antiviral Treatment	Route of Administration	Associated Adverse Effects	Outcome
Adler H, et al. [18]	1	Skin lesions, lymphadenopathy, fever, and night sweats	Face, scalp, trunk, limbs, palms, glans penis, and scrotum	Brincidofovir 200 mg (one dose)	Oral	Transaminitis	Full recovery
	2	Skin lesions, lymphadenopathy, fever, and groin swelling	Face, trunk, limbs, palms, soles, and scrotum	Brincidofovir 200 mg (two doses)	Oral	Transaminitis	Full recovery
	3	Skin lesions and coryzal illness	Face, trunk, hands (including nail bed), and labia majora	Brincidofovir 200 mg (two doses)	Oral	Transaminitis, nausea, and abdominal discomfort	Full recovery
	4	Skin lesions, lymphadenopathy, fever, and headache	Face, scalp, trunk, limbs, penile shaft, palms, and soles	None	None	None	Full recovery
	5	Skin lesions and lymphadenopathy	Face, trunk, limbs, palms, and penile shaft	None	None	None	Full recovery
	6	Skin lesions and lymphadenopathy	Face, trunk, arms, and legs	None	None	None	Full recovery
	7	Skin lesions	Face, trunk, arms, and hands	Tecovirimat 600 mg twice daily for 2 weeks	Oral	None	Full recovery
Desai AN, et al. [19]	25	Skin lesions (*n* = 25), fever (*n* = 19), lymphadenopathy (*n* = 13), headache (*n* = 8), fatigue (*n* = 7), sore throat (*n* = 5), chills (*n* = 5), back pain (*n* = 3), myalgia (*n* = 2), nausea (*n* = 1), and diarrhea (*n* = 1).	Genital and/or perianal (*n* = 23), chest (*n* = 9), arms (*n* = 13), back (*n* = 8), face (*n* = 7), and legs (*n* = 6).	Tecovirimat every 8 or 12 h for 2 weeks (*n* = 25)	Oral	Fatigue (*n* = 7), headache (*n* = 5), nausea (*n* = 4), itching (*n* = 2), and diarrhea (*n* = 2)	Full recovery
Matias WR, et al. [20]	1	Skin lesions, lymphadenopathy, fever, chills, and general malaise.	Penis, pubis, and arm	Tecovirimat 600 mg twice daily for 2 weeks	Oral	Transaminitis, headache	Full recovery
	2	Skin lesions, lymphadenopathy, fever, chills, myalgias, left tonsillar pain, and odynophagia	Forearms and hands	Tecovirimat 600 mg twice daily for 2 weeks	Oral	Liquid stools	Full recovery
	3	Skin lesions, lymphadenopathy, malaise, and subjective fevers	Penis, chest, and arm	Tecovirimat 600 mg twice daily for 2 weeks	Oral	None	Full recovery
Thornhill JP, et al. [1]	528	Rash or skin lesions (*n* = 500), fever (*n* = 330), lymphadenopathy (*n* = 295), lethargy or exhaustion (*n* = 216), myalgia (*n* = 165), headache (*n* = 145), pharyngitis (*n* = 113), low mood (*n* = 54), and proctitis or anorectal pain (*n* = 75).	Anogenital area (*n* = 383), trunk or limbs (*n* = 292), face (*n* = 134), palms or soles (*n* = 51), and mucosal lesions (*n* = 217).	Cidofovir (*n* = 12), tecovirimat (*n* = 8), vaccinia immune globulin (*n* = 1)	Oral and parenteral	NR	Full recovery
Moschese D, et al. [21]	1	Skin lesions, fever, chills, sweats, and lymphadenopathy	Nose, limb	Cidofovir 5 mg/kg day 1 and 7	Intravenous	None	Full recovery
	2	Skin lesions, fever, and lymphadenopathy	Head, limbs, and trunk	None	NR	None	Full recovery
	3	Skin lesions, fever, and lymphadenopathy	Perianal, foot, face, and arm	None	NR	None	Full recovery
	4	Skin lesions, fever, headache, and lymphadenopathy	Inguinal, penis, scrotum, and face	None	NR	None	Full recovery
Rao AK, et al. [22]	1	Purulent rash, diarrhea, vomiting, cough, subjective fever, and fatigue	NR	Tecovirimat	Oral	None	Full recovery
Mailhe M, et al. [23]	264	Skin lesions (*n* = 264), lymphadenopathy (*n* = 174), fever (*n* = 171), pharyngitis (*n* = 51), angina (*n* = 41), respiratory signs (*n* = 31), and headaches (*n* = 89)	Genital area (*n* = 135), limbs (*n* = 121), trunk (*n* = 105), perianal (*n* = 100), face (*n* = 88), and palmoplantar area (*n* = 36)	Two doses of Cidofovir 5 mg/kg. (*n* = 1)	Intravenous	None	Full recovery
Minhaj, F.S. et al. [24]	17	Skin lesions (*n* = 17), fatigue or malaise (*n* = 13), chills (*n* = 12), lymphadenopathy (*n* = 9), headache (*n* = 8), fever (*n* = 7), body aches (*n* = 6), sore throat or cough (*n* = 5), and sweat (*n* = 4).	Arm (*n* = 9), trunk (*n* = 9), legs (*n* = 8), face (*n* = 7), hands (*n* = 6), perianal (*n* = 6), oral (*n* = 5), neck (*n* = 5), genital (penis or vagina) (*n* = 4), and feet (*n* = 4).	Tecovirimat (*n* = 1)	Oral	None	Full recovery
Girometti, N. et al. [25]	54	Skin lesions (*n* = 54), Fatigue (*n* = 36), fever (*n* = 31), lymphadenopathy (*n* = 30), myalgia (*n* = 16), and sore throat (*n* = 11)	Genital (*n* = 33), perianal (*n* = 24), upper and lower extremities (*n* = 27), facial (*n* = 11), oropharyngeal (*n* = 4), and torso (*n* = 14)	Tecovirimat (*n* = 1).	Oral	None	Full recovery
Tarín-Vicente, E.J. et al. [26]	181	Skin lesions (*n* = 181), lymphadenopathy (*n* = 153), Influenza-like illness (*n* = 147), fever (*n* = 131), headache (*n* = 96), and sore throat (*n* = 66)	Genital (*n* = 100), perianal area (*n* = 66), oral ulcer (*n* = 45), perioral (*n* = 51), hands and feet (*n* = 108), trunk and extremities (*n* = 104)	Cidofovir (*n* = 6)	Cutaneous	None	Full recovery
Patel, A. et al. [27]	197	Mucocutaneous manifestations (*n* = 197), fever (*n* = 122), lymphadenopathy (*n* = 114), headache (*n* = 49), fatigue/lethargy (*n* = 46), myalgia (*n* = 62), arthralgia (*n* = 21), back pain (*n* = 21), and rectal pain or pain on defecation (*n* = 71)	Face (*n* = 71), trunk (*n* = 70), arms/legs (*n* = 74), hands/feet (*n* = 56), genitals (*n* = 111), anus or perianal area (*n* = 82), and oropharyngeal (*n* = 27)	Tecovirimat 600 mg twice daily for 14 days (*n* = 1).	Oral	None	Full recovery

NR: No report.

## Data Availability

Available upon reasonable request.
